# The failure of glomerular filtration rate estimating equations among obese population

**DOI:** 10.1371/journal.pone.0242447

**Published:** 2020-11-18

**Authors:** Piyawan Kittiskulnam, Krittaya Tiskajornsiri, Pisut Katavetin, Tawatchai Chaiwatanarat, Somchai Eiam-Ong, Kearkiat Praditpornsilpa

**Affiliations:** 1 Division of Internal Medicine-Nephrology, Department of Medicine, Faculty of Medicine, Chulalongkorn University and King Chulalongkorn Memorial Hospital, Thai Red Cross Society, Bangkok, Thailand; 2 Special Task force for Activating Research in Renal Nutrition (Renal Nutrition Research Group), Office of Research Affairs, Chulalongkorn University, Bangkok, Thailand; 3 Division of Nephrology, Department of Medicine, Faculty of Medicine, Chulalongkorn University, Bangkok, Thailand; 4 Division of Nephrology, Banpaeo Hospital, Samutsakorn, Thailand; 5 Division of Nuclear Medicine, Department of Radiology, Faculty of Medicine, Chulalongkorn University, Bangkok, Thailand; Istituto Di Ricerche Farmacologiche Mario Negri, ITALY

## Abstract

**Background:**

Obesity is a major public health with increasing numbers of obese individuals are at risk for kidney disease. However, the validity of serum creatinine-based glomerular filtration rate (GFR) estimating equations in obese population is yet to be determined.

**Methods:**

We evaluated the performance of the reexpressed Modification of Diet in Renal Disease (MDRD), reexpressed MDRD with Thai racial factor, Thai estimated GFR (eGFR) as well as Chronic Kidney Disease Epidemiology Collaboration (CKD-EPI) equations among obese patients, defined as body mass index (BMI) ≥25 kg/m^2^ with the reference measured GFR (mGFR) determined by ^99m^Tc-diethylene triamine penta-acetic acid (^99m^Tc-DTPA) plasma clearance method. Serum creatinine levels were measured using standardized enzymatic method simultaneously with GFR measurement. The statistical methods in assessing agreement for continuous data including total deviation index (TDI), concordance correlation coefficient (CCC), and coverage probability (CP) for each estimating equation were compared with the reference mGFR. Accuracy within 10% representing the percentage of estimations falling within the range of ±10% of mGFR values for all equations were also tested.

**Results:**

A total of 240 Thai obese patients were finally recruited with mean BMI of 31.5 ± 5.8 kg/m^2^. In the total population, all eGFR equations underestimated the reference mGFR. The average TDI values were 55% indicating that 90% of the estimates falling within the range of -55 to +55% of the reference mGFR. The CP values averaged 0.23 and CCC scores ranged from 0.75 to 0.81, reflecting the low to moderate levels of agreement between each eGFR equation and the reference mGFR. The proportions of patients achieving accuracy 10% ranged from 23% for the reexpressed MDRD equation to 33% for the Thai eGFR formula. Among participants with BMI more than 35 kg/m^2^ (n = 48), the mean error of all equations was extremely wide and significantly higher for all equations compared with the lower BMI category. Also, the strength of agreement evaluated by TDI, CCC, and CP were low in the subset of patients with BMI ≥35 kg/m^2^.

**Conclusion:**

Estimating equations generally underestimated the reference mGFR in subjects with obesity. The overall performance of GFR estimating equations demonstrated poor concordance with the reference mGFR among individuals with high BMI levels. In certain clinical settings such as decision for dialysis initiation, the direct measurements of GFR are required to establish real renal function among obese population.

## Introduction

The rise of obesity prevalence, defined by body mass index (BMI) criteria, continues to increase worldwide not only in developed but also in developing countries [1]. Monitoring of trends using data from the Thai National Health Examination Surveys has shown a secular trend as the prevalence of obesity in adults has been almost doubled in the past decades [2]. Obesity can directly impact the kidney through the development of hyperfiltration-related glomerulomegaly, adipokine-associated inflammation, and insulin resistance. Furthermore, other adverse renal consequences of obesity may be mediated by its accompanying comorbid conditions such as hypertension and diabetes [3]. Therefore, obesity has become a major health challenge with increasing numbers of individuals are at risk for kidney disease. Previous studies reported that a high BMI ranks as one of the strongest predictors for developing the new-onset chronic kidney disease (CKD) in general population [4, 5]. Also, elevated BMI levels have been associated with more rapid loss of glomerular filtration rate (GFR) over time among patients with pre-existing CKD [6]. However, the methods of assessing kidney function either for CKD classification or drug dosing in obese population are still unestablished.

Although the evaluation of kidney function by measured GFR (mGFR) using exogenous filtration marker clearance is considered as a highly accurate method, the measurement protocol is somewhat complicated. Currently, the Kidney Disease Improving Global Outcomes (KDIGO) guideline recommends using calibrated serum creatinine-based equations to estimate kidney function and estimated GFR (eGFR) should be reported in relative to body surface area (BSA) of 1.73 m^2^ in adults [7]. The original Modification of Diet in Renal Disease (MDRD) equation was subsequently adjusted as the “reexpressed” MDRD equation after standardizing with traceable high-level isotope dilution mass spectroscopy (IDMS) reference serum creatinine level measured by enzymatic method [8, 9]. To account for possible differences in muscle mass as well as diet according to ethnicity and geographic region, the MDRD equation has been modified for using in racial groups other than Caucasian and African-American [10–12]. In 2009, the Chronic Kidney Disease Epidemiology (CKD-EPI) Collaboration equation was introduced utilizing a stepwise multiple regression to determine a subset of variables to predict GFR from pooled CKD and non-CKD participants to improve limitations of the MDRD equation in patients with GFR ≥ 60 mL/min/1.73m^2^ [13]. Nonetheless, neither body weight nor height as a determination of body size was finally included in the 4-variable model (age, sex, race, and serum creatinine) of the reexpressed MDRD formula as well as the CKD-EPI equation.

The agreement of eGFR estimated by the MDRD and CKD-EPI equations with the reference mGFR has been externally validated in diverse populations with the majority of participants having normal BMI levels [14–16]. However, only few studies, all of which were limited to Caucasians, have formerly assessed GFR prediction equations among obese population [17, 18]. Whether the validity of available GFR estimating equations indexed to BSA could be used in individuals with larger body size than the reference non-obese populations is yet to be determined. In the present study, we compared the overall performance of eGFR derived from the reexpressed MDRD, the reexpressed MDRD with Thai racial factor, the Thai eGFR, and the CKD-EPI equations with the mGFR using the gold standard reference method of the ^99m^Tc-diethylene triamine pentaacetic acid (^99m^Tc-DTPA) plasma clearance among Thai obese population.

## Materials and methods

### Study design and participants

This was a cross-sectional study conducted from March 2012 to December 2018. Participants were recruited from our tertiary care outpatient-clinic at King Chulalongkorn Memorial Hospital, Bangkok, Thailand. Eligible participants were over 18 years old with diagnosis of obesity, defined as BMI ≥ 25 kg/m^2^ among Asian population according to the International Obesity Task Force criteria [19], had stable condition, and were able to provide inform consent. The patients’ medical records were extracted to collect information regarding their current medication prescription, laboratory results, and comorbid conditions. Participants with acute deterioration of kidney function, amputation, severe malnutrition, concurrent active infection, gastrointestinal bleeding, congestive heart failure, uncontrolled edematous state, pregnancy, and having a history of radioisotope hypersensitivity were excluded. Treatment with corticosteroids, ascorbic acid, methyldopa, flucytosine, cimetidine, or trimethoprim were not allowed. Body composition was also assessed by multi-frequency bioelectrical impedance analysis (InBodyS20^®^, Biospace Corp., Seoul, Korea). The study was performed in compliance with the Helsinki Declaration. All participants were informed and provided written informed consent to participate in this study. The study was approved by the Ethical Committee for Research at the Faculty of Medicine, Chulalongkorn University, Bangkok, Thailand (IRB No.355/54).

### Measurement of reference GFR

The reference mGFR was measured by collecting plasma sample from ten different time points using more than 95% radiopurity and less than 5% plasma protein bound ^99m^Tc-DTPA at Division of Nuclear Medicine, Department of Radiology, Chulalongkorn University. This radioactive substance was procured from the Office of Atoms for Peace, Bangkok, Thailand. The ^99m^Tc-DTPA plasma clearance method was performed at 8.00–9.00 AM to avoid the diurnal variations in renal function and a single intravenous bolus with a dose of 3 millicurie was injected in 5–10 minutes. Blood specimens were drawn to assess plasma radioactivity at 5, 10, 20, 30, 60, 90, 120, 180, and 240 minute post injection using heparin lock and were then plotted as a function of time to create a time-activity curve to derive GFR. The reference mGFR values were evaluated by a blind radiologist to the patients’ clinical data. The repeated protocol was applied to all patients. The reference mGFR was determined by using bi-exponential fitting method calculated by dosage of ^99m^Tc-DTPA and area under time-activity curve [20] and was normalized to BSA utilizing the most commonly used Du Bois formula [21].

### Estimating of serum creatinine-based GFR equations

Fasting serum creatinine was sampled on the same day as GFR measurement by using Roche Diagnostics (Indianapolis, IN) CREA Plus (11775642) enzymatic assay on a COBAS INTEGRA^®^ 400 plus analyzer. All measured enzymatic creatinine values were then calibrated by using traceable high-level IDMS reference serum creatinine, as recommended by the National Kidney Disease Education Program [22]. The IDMS reference serum creatinine (SRM 967) was derived from the National Institute of Standards and Technology. The certified concentration values of serum creatinine were 0.847 ± 0.018 mg/dL for level 1 and 3.877 ± 0.082 mg/dL for level 2. The GFR values were then estimated by using the reexpressed MDRD, the reexpressed MDRD with Thai racial factor correction of 1.129, the Thai eGFR formula [10], and the CKD-EPI equation and expressed as a unit of mL/min/1.73m^2^, as summarized in **Table 1**.

**Table 1:** Estimating GFR equations.

****eGFR methods****	****Gender****	****Serum creatinine****	****Equations****
Reexpressed MDRD [8]	Male	Cr_Enz_	175 × (Cr_Enz_)^− 1.154^ × (Age)^− 0.203^
	Female		175 × (Cr_Enz_)^− 1.154^ × (Age)^− 0.203^ × 0.742
Reexpressed MDRD with Thai racial factor [10]*	Male	Cr_Enz_	175 × (Cr_Enz_)^ −1.154^ × (Age)^−0.203^ × 1.129
	Female		175 × (Cr_Enz_)^ −1.154^ × (Age)^−0.203^ × 1.129 × 0.742
Thai eGFR formula [10]^†^	Male	Cr_Enz_	375.5 × (Cr_Enz_) ^−0.848^ × (Age)^−0.364^
	Female		375.5 × (Cr_Enz_) ^−0.848^ × (Age)^−0.364^ × 0.712
CKD-EPI [13]	Male	Cr_Enz_ ≤ 0.9 mg/dL	141 × (Cr_Enz_/0.9)^− 0.411^ × (0.993)^Age^
	Male	Cr_Enz_ > 0.9 mg/dL	141 × (Cr_Enz_/0.9)^− 1.209^ × (0.993)^Age^
	Female	Cr_Enz_ ≤ 0.7 mg/dL	144 × (Cr_Enz_/0.7)^− 0.329^ × (0.993)^Age^
	Female	Cr_Enz_ > 0.7 mg/dL	144 × (Cr_Enz_/0.7)^− 1.209^ × (0.993)^Age^

Cr_Enz_, serum creatinine assay measured by enzymatic method; CKD-EPI, Chronic Kidney Disease Epidemiology; MDRD, Modification of Diet in Renal Disease.

*The coefficient for Thai racial factor was 1.129 for reexpressed MDRD equation.

^†^ The Thai eGFR formula obtained from variables that could predict GFR among Thais.

### Statistical analysis

We described patient characteristics using mean ± standard deviation (SD) for normally distributed or median (25^th^ - 75^th^ percentile) for non-normally distributed variables and proportions for categorical variables. Bias or mean error was defined as the mean difference between eGFR and reference mGFR. Precision was expressed as the degree of scatter in a series of eGFR values (SD of bias). The agreement of various eGFR equations with the reference mGFR were assessed using the limit of agreement, total deviation index (TDI), concordance correlation coefficient (CCC), coverage probability (CP). The limit of agreement is a range that encompasses most differences between eGFR and mGFR with the reference interval defined as mean ± 1.96 x 1SD [23]. TDI is a measure that captures a large proportion of data within a boundary representing the allowable difference between two measurements. Empirical TDI was calculated for a theoretical TDI of 10% and a CP of 90%. The ideal situation would be a TDI of <10%, meaning that 90% of eGFR values fall within ± 10% of the reference mGFR. The CP values range from 0 to 1, and estimates whether a given TDI is less than a pre-specified fixed percentage [24]. The CCC scores combines the elements of accuracy and precision (range 0–1). A CCC values > 0.9 reflects optimal concordance between measurements. Accuracy was described as the degree of closeness of eGFR to the mGFR which was calculated as the percentage of GFR estimations falling within the range of 10% and 30% below or above (± 10% and ± 30%) of mGFR values for each estimating equation, respectively. Bland-Altman plots were also used to assess the agreement between eGFR and the reference mGFR. We conducted all analyses using the statistical package Agreement Program (AGP v1.0, IGEKO, SP) [25] which is based on the R code developed by Lin *et al*. [24] and available at www.ecihucan.es/lfr/apps/?dir=agreement_installer. We also analyzed that data using Stata 15 (StataCorp LP, College Station, TX), and *P* values less than 0.05 were considered statistically significant.

## Results

### Baseline demographic data of participants

A total of two hundred and forty patients were finally recruited in the study. The mean age of patients was 52.4 ± 15.2 years with 40% men and 35.1% diabetes. The average BSA and BMI were 1.8 ± 0.2 m^2^ and 31.5 ± 5.8 kg/m^2^, respectively. Total percent body fat was significantly higher in women than in men (45.9 ± 5.2 *vs* 35.6 *±* 5.6%, *p*<0.001). Demographic characteristics including age, sex, diabetes, BMI, and laboratory data of the study population are shown in **Table 2**. The average reference mGFR and serum creatinine were 89.3 ± 52.5 mL/min/1.73m^2^ and 1.5 ± 1.3 mg/dL, respectively. The mean reference mGFR were not significantly different between both sexes (85.2 ± 57.6 mL/min/1.73m^2^ in men and 92.0 ± 48.8 mL/min/1.73m^2^ in women, *p* = 0.33).

**Table 2:** Patient characteristics at baseline.

****Parameters****	****Total (n = 240)****	****Male (n = 96)****	****Female (n = 144)****	****P value****
Age, years	52.4 ± 15.2	55.1 ± 15.3	50.7 ± 14.9	0.03
Body weight, kg	81.6 ±17.5	82.3 ± 14.8	81.1 ± 19.2	0.60
Height, m	1.6 ± 0.1	1.7 ± 0.1	1.6 ± 0.1	<0.001
BSA, m^2^	1.8 ± 0.2	1.9 ± 0.2	1.8 ± 0.2	0.001
BMI, kg/m^2^	31.5 ± 5.8	30.0 ± 5.0	32.5 ± 6.1	0.001
BMI ≥30, %	51.3	33.3	63.2	<0.001
BMI ≥35, %	20.0	13.5	24.3	0.04
BMI ≥40, %	7.9	5.2	9.7	0.21
Percent body fat*, %	43.1 ± 7.0	35.6 ± 5.6	45.9 ± 5.2	<0.001
Diabetes, %	36.7	42.7	32.8	0.15
Hypertension, %	58.6	64.6	54.7	0.15
Systolic blood pressure, mmHg	133.0±21.9	132.8±21.6	133.2±22.2	0.89
Diastolic blood pressure, mmHg	81.7±11.7	81.8±13.4	81.7±10.6	0.98
Serum creatinine, mg/dL	1.5±1.3	1.9±1.6	1.1±0.8	<0.001
Blood urea nitrogen, mg/dL	15.6 (12.1–25.3)	18.1 (13.9–29.9)	13.8 (11.0–22.7)	0.001
Measured GFR, ml/min	96.9 ± 59.1	95.2 ± 65.0	98.1 ± 55.1	0.71
Measured GFR, ml/min/1.73m^2^	89.3 ± 52.5	85.2 ± 57.6	92.0 ± 48.8	0.33
GFR <90, %	52.5	64.6	44.4	0.002
GFR <60, %	34.2	40.6	29.9	0.08
GFR <30, %	12.5	11.5	13.2	0.69
Albumin, g/dL	4.2 ± 0.4	4.3 ± 0.4	4.1 ± 0.4	0.001
Uric acid, g/dL	6.3 (5.1–7.8)	7.5 (6.1–8.7)	6.0 (4.7–7.2)	0.001
Total cholesterol, mg/dL	194.8 ± 52.3	185.0 ± 49.8	200.9 ± 53.1	0.03
Triglyceride, mg/dL	156.7 ± 91.3	165.4 ± 100.9	151.1 ± 84.5	0.26
Urine protein creatinine ratio, g/day	0.8 ± 1.5	0.9 ± 1.5	0.8 ± 1.5	0.71

BMI, body mass index; BSA, body surface area; GFR, glomerular filtration rate.

Data are presented as mean ±SD and median (25^th^ - 75^th^).

*P*<0.05 consider significantly different between two groups.

*Complete data available for 106 patients.

### Comparison of agreement analyses among eGFR equations

**Fig 1:**
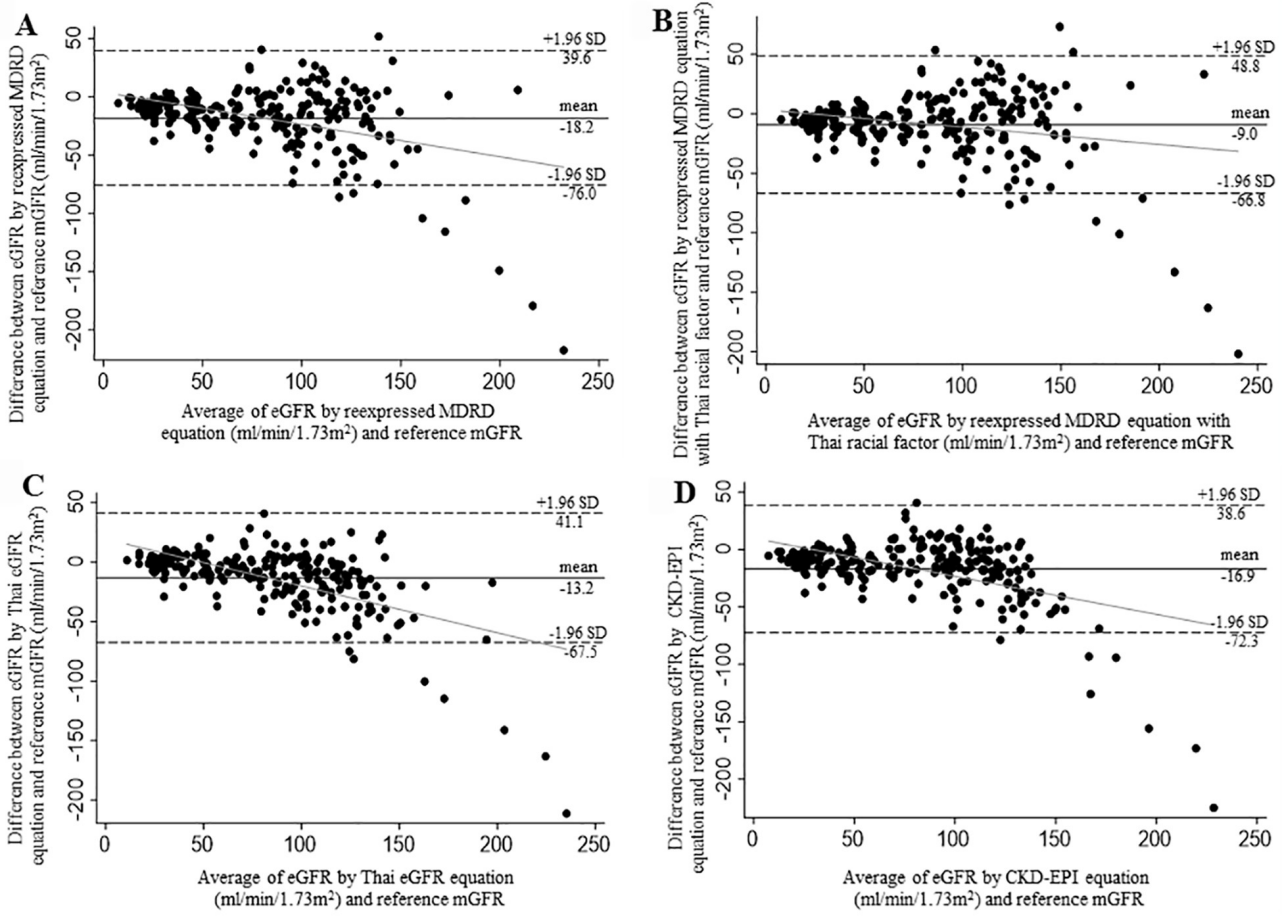
Bland-Altman plots for eGFR by different equations and the reference mGFR. 1A: Reexpressed MDRD equation; 1B: Reexpressed MDRD equation with Thai racial factor; 1C: Thai eGFR equation; 1D: CKD-EPI equation. The continuous black line represents the mean difference between eGFR and mGFR. The dashed line represents the limits of agreement (mean difference ± 1.96 SD). The continuous grey line represents the regression trend of mean difference and the average of eGFR and mGFR.

In the total participants, all estimating equations underestimated the reference mGFR. The mean error (bias) and precision of the reexpressed MDRD equation (-18.2 ± 29.5 mL/min/1.73m^2^) were significantly different from the reexpressed MDRD with Thai racial factor (-9.0 ± 29.5 mL/min/1.73m^2^, *p*<0.001) as well as the Thai eGFR formula (-13.2 ± 27.7 mL/min/1.73m^2^, *p*<0.001) but not the CKD-EPI equation (-16.9 ± 28.3 mL/min/1.73m^2^, *p* = 0.34). The analyses of Bland-Altman plot for each eGFR equation revealed wide limits of agreement with the reference mGFR (**Fig 1**). The average TDI values were 55% among all equations indicating that 90% of the estimates falling within the range of -55 to +55% of the reference mGFR (**Table 3**).

**Table 3:** Comparison of performance of all eGFR equations with the reference measured GFR.

Parameters	****Reference mGFR (**^**99m**^**Tc-DTPA)****	****Reexpressed MDRD equation****	****Reexpressed MDRD with Thai racial factor****	****Thai eGFR equation****	****CKD-EPI equation****
Total (n = 240)					
Mean GFR (ml/min/1.73m^2^)	89.3 ± 52.5	71.3 ± 40.8	80.5 ± 46.1	76.2 ± 36.2	72.5 ± 38.5
CCC	NA	0.75	0.81	0.78	0.76
TDI	NA	57.0	50.7	50.6	54.2
CP	NA	0.22	0.25	0.25	0.23
Accuracy 10%	NA	23.4	26.8	33.1	23.8
Accuracy 30%	NA	63.2	74.9	84.5	70.7
mGFR ≥ 60 ml/min/1.73m^2^ (n = 158)					
Mean GFR (ml/min/1.73m^2^)	116.8 ± 43.6	94.3 ± 29.6	106.5 ± 33.4	97.0 ± 25.1	95.8 ± 23.5
CCC	NA	0.48	0.57	0.53	0.47
TDI	NA	68.4	60.8	61.6	64.9
CP	NA	0.18	0.21	0.20	0.19
Accuracy 10%	NA	27.4	27.4	34.4	28.7
Accuracy 30%	NA	70.1	79.0	82.8	80.3
mGFR < 60 ml/min/1.73m^2^ (n = 82)					
Mean GFR (ml/min/1.73m^2^)	36.5 ± 12.1	27.0 ± 13.9	30.5 ± 15.7	36.2 ± 13.1	27.6 ± 14.0
CCC	NA	0.57	0.65	0.79	0.59
TDI	NA	22.3	20.1	13.4	21.5
CP	NA	0.48	0.57	0.78	0.51
Accuracy 10%	NA	18.8	25.6	30.5	14.6
Accuracy 30%	NA	50.0	67.1	87.8	52.4

CCC, concordance correlation coefficient; CP, coverage probability, CKD, chronic kidney disease; mGFR, measured glomerular filtration rate; NA, not applicable; TDI, total deviation index; ^99m^Tc-DTPA, ^99m^Tc-diethylene triamine penta-acetic acid.

The CP values ranged from 0.22 to 0.25, meaning that more than 75–78% of obese subjected had an error greater than ±10% of the reference mGFR. The CCC scores for the reexpressed MDRD, the reexpressed MDRD with Thai racial factor, the Thai eGFR formula, and the CKD-EPI equations were 0.75 [95% confident interval, CI; 0.69–0.79], 0.81 [95% CI; 0.76–0.85], 0.78 [95% CI; 0.74–0.82], and 0.76 [95% CI; 0.71–0.80], respectively, representing the low to moderate level of agreement between each eGFR equation and the reference mGFR. Thirty-four percent of patients had reference mGFR below 60 mL/min/1.73m^2^ (n = 82). Regarding the different mGFR categories, all equations demonstrated poor concordance in terms of TDI, CCC, and CP values and these discrepancies were comparable among eGFR equations with or without specific racial modification (**Table 3**). Of particular, none of the estimating equations yielded TDI values of less than 10% among patients having mGFR levels < 60 mL/min/1.73m^2^.

### Performance on accuracy among estimating equations

The Thai eGFR equation had more accuracy within 30% when compared with the reexpressed MDRD equation (*p*<0.001), the reexpressed MDRD with Thai racial factor (*p* = 0.003), and the CKD-EPI equations (*p*<0.001). For estimating equations without specific racial modification, the CKD-EPI equation demonstrated better accuracy compared to the reexpressed MDRD equation (70.7 *vs* 63.2%, *p* = 0.03). However, the loss of accuracy within 10% were identified among all estimating equations (**Table 3**). The proportion of patients having eGFR results within ± 10% of the reference mGFR were only 23.4, 26.8, 33.1, and 23.8% for the reexpressed MDRD, the reexpressed MDRD with Thai racial factor, the Thai eGFR, and the CKD-EPI equations, respectively.

### Performance of different equations among BMI subgroups

**Fig 2:**
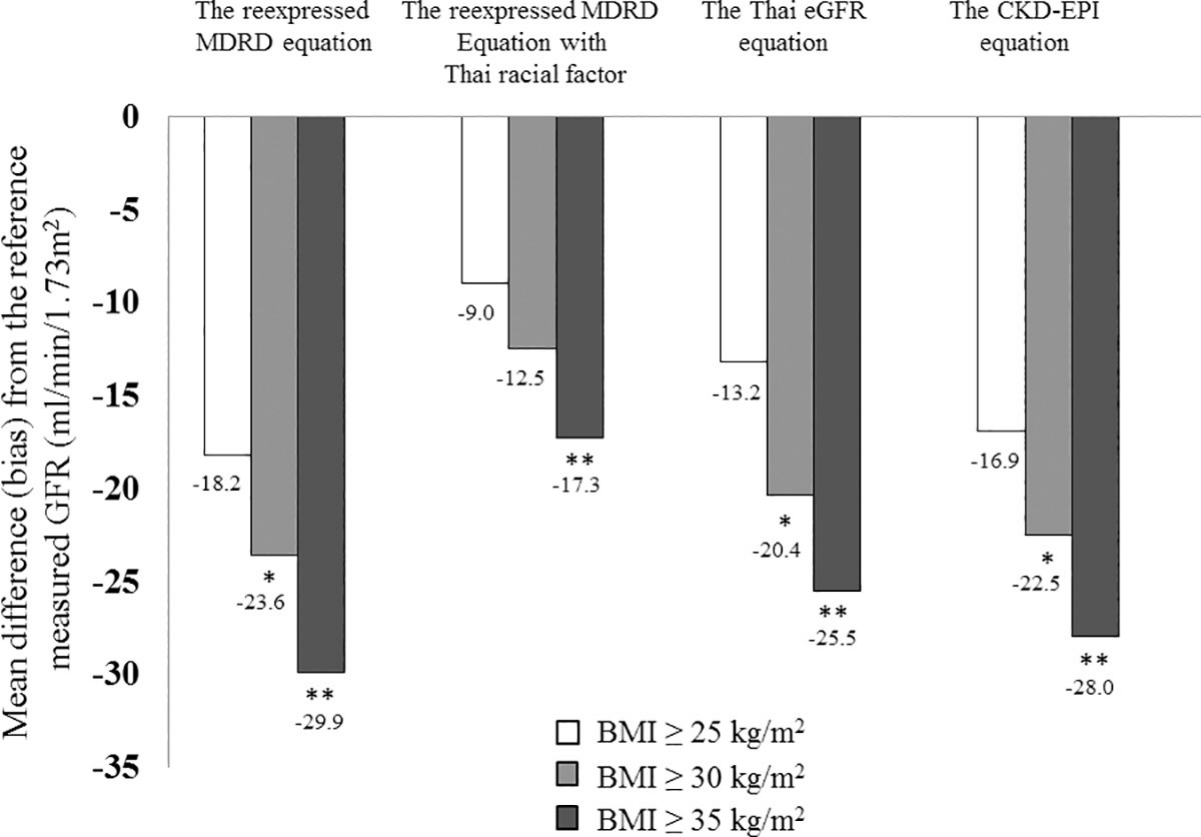
Mean difference (bias) of each estimating equation from the reference mGFR according to BMI subgroups. *denotes *p* values less than 0.05 between BMI ≥30 and 25–29.9 kg/m^2^ for each estimating eGFR equation. **denotes *p* values less than 0.05 between BMI ≥ 35 and 25–34.9 kg/m^2^ for each estimating eGFR equation.

There were 123 patients with BMI more than 30 kg/m^2^, the level utilized to define obesity in Caucasians, with the mean BMI of 35.4 ± 5.7 kg/m^2^. The mean error of all estimating equations were higher compared to their lower BMI levels and increased with BMI categories (**Fig 2**). Forty-eight patients were classified as having BMI more than 35 kg/m^2^ with the average BMI of 40.6 ± 6.1 kg/m^2^. The mean difference between eGFR and reference mGFR of all equations was extremely wide. The relatively low strength of agreements among each eGFR equation and the reference mGFR were repeatedly observed with the TDI values of 66.7 to 77.0% and the CCC scores of 0.51–0.61. Furthermore, the percentage of patients having the accuracy within 10% were unacceptably low ranging from 16.7% for the reexpressed MDRD equation to 29.2% by the Thai eGFR formula (**Table 4**).

**Table 4:** Comparison of performance of all eGFR equations with reference measured GFR according to different BMI subgroups.

Parameters	****Reference mGFR (**^**99m**^**Tc-DTPA)****	****Reexpressed MDRD equation****	****Reexpressed MDRD with Thai racial factor****	****Thai eGFR equation****	****CKD-EPI equation****
BMI ≥ 30 kg/ m^2^ (n = 123)					
CCC	NA	0.64	0.72	0.67	0.65
TDI	NA	71.4	63.2	65.3	68.4
CP	NA	0.18	0.20	0.19	0.18
Accuracy 10%	NA	24.6	24.6	28.7	26.2
Accuracy 30%	NA	65.6	75.4	78.7	72.9
BMI ≥ 35 kg/ m^2^ (n = 48)					
CCC	NA	0.51	0.61	0.57	0.54
TDI	NA	77.0	66.7	68.2	70.9
CP	NA	0.16	0.19	0.18	0.17
Accuracy 10%	NA	16.7	18.7	29.2	27.1
Accuracy 30%	NA	68.7	70.8	77.1	72.9

BMI, body mass index; CCC, concordance correlation coefficient; CP, coverage probability, CKD, chronic kidney disease; mGFR, measured glomerular filtration rate; NA, not applicable; TDI, total deviation index; ^99m^Tc-DTPA, ^99m^Tc-diethylene triamine penta-acetic acid.

## Discussion

In Thai obese subjects defined by BMI ≥ 25 kg/m^2^, the results in the present study demonstrated that GFR estimation by using the reexpressed MDRD, the reexpressed MDRD with Thai racial factor, the Thai eGFR as well as the CKD-EPI equations generally underestimated the reference GFR measurement determined by ^99m^Tc-DTPA plasma clearance method. The overall performance of GFR estimating equations either on the strength of agreement or accuracy compared with the reference mGFR were poor among our obese population.

The frequently used creatinine-based eGFR equations typically yield some bias estimates given that pre-existing GFR prediction equations are developed using regression analysis to correlate the reference mGFR to steady state serum creatinine concentration and surrogates of non-GFR determinants of serum creatinine [7]. The reexpressed MDRD without racial factor and CKD-EPI formulas were developed mainly in non-obese European black and white descent and, thus might not be suitable for estimating GFR in obese Asian population. In the present study with higher mean participants’ BMI than a previous study using the MDRD equation of 27.0 ± 5.0 kg/m^2^ [26] and the CKD-EPI study of 28.0 ± 6.0 kg/m^2^ [13], we demonstrated that all eGFR equations generally underestimated the reference mGFR (**Fig 2**). Using similar ^99m^Tc-DTPA as the reference mGFR and IDMS-traceable serum creatinine concentration, an earlier study by Chew-Harris *et al*. [27] among true obesity subjects defined as BMI ≥ 30 kg/m^2^ combined with percent body fat ≥ 30% supported our study that the MDRD and CKD-EPI equations underestimated the reference mGFR of -18.6 and -11.8 mL/min/1.73m^2^, respectively. On the other hand, findings from the study of Friedman *et al*. [28] among severely obese patients with pre-bariatric surgery BMI of 46 kg/m^2^ indicated that the average differences from the reference mGFR determined by plasma iohexol clearance were overestimated approximately +7.0 mL/min/1.73m^2^ for MDRD and +13.5 mL/min/1.73m^2^ for CKD-EPI equations. This discrepancy could be partially explained by the fact that indexing GFR with per 1.73 m^2^ of BSA might result in substantially lower GFR than unindexed GFR in the setting of obesity [29]. Indeed, the increased BSA had less effect on enhanced creatinine excretion than the elevated BMI when BMI was more than 30 kg/m^2^ [30]. Therefore, at very high BMI extreme such as morbid obesity, equations that standardized eGFR to BSA might overestimate the reference mGFR (**Table 1**).

The differences in ethnicity can significantly affect the results obtained from the MDRD-based eGFR equation. Either a development of ethnicity factor or a separate eGFR equation would optimize the regional estimation of GFR and, at least, have applicability for the specific local CKD patient population [31, 32]. In the current study, the Thai eGFR equation had more accuracy within 30% when compared with the reexpressed MDRD equation, the reexpressed MDRD with Thai racial factor, and the CKD-EPI equations (all *p*<0.01). Although previous studies in obese European population [18] and most Caucasians participants with BMI 30–35 kg/m^2^ [33] reported that the CKD-EPI equation did not outperform the MDRD study equation with regard to the accuracy of GFR estimation, our findings consistently showed that the CKD-EPI equation demonstrated better accuracy within 30% compared to the reexpressed MDRD equation in both the whole study population and BMI subgroup of more than 30 kg/m^2^. In accordance with the present study but having slightly lower participants’ BMI level, Jessani and colleagues [34] found that the CKD-EPI equation was more accurate than the MDRD equation among South Asian population. Moreover, the results from the Swedish cohort [35] were similar to ours, but having lower proportion of subjects with BMI ≥ 30 kg/m^2^, that the accuracy of the CKD-EPI equation was substantially higher than the reexpressed MDRD equation at higher compared to lower GFR levels.

Although the Kidney Disease Outcomes Quality Initiative guideline recommendations have suggested that 75% of eGFR has to be within ± 30% of mGFR to be considered as sufficient for good clinical decision making [36], the accuracy within 30% in this context was defined in the absence of any clinical rationale as an acceptable margin of error. In recognition that the variability of GFR measurement by various methods occurred from the intrinsic error of methods and the biological variability of GFR is approximately 4–8%, an acceptable limit of agreement between eGFR and mGFR should not exceed ±10% of mGFR and 90% of estimations should fall within this margin of error [37]. When assessing with more appropriate statistical approaches including accuracy within 10% and agreement analyses using TDI, CCC, CP values, our results revealed that all GFR estimating equations demonstrated poor concordance with the reference mGFR. In general, the CKD-EPI equation which were developed in a pooled population covering the entire spectrum of CKD and non-CKD participants are considered more reliable than the previous MDRD equation which was derived from CKD patients [7]. However, a single estimating equation is unlikely to work equally well in all populations. In fact, all equations for GFR estimation are essentially mathematical abstractions that correlate patients to the selected populations from which the estimating equations were derived. Therefore, it is strongly recommended that one should bear in mind regarding the potential limitations for the use of GFR estimating equations in real clinical applications.

In the setting of extreme obesity, the validity of estimating equations indexed to BSA obtained from non-obese individuals as reference population was controversial. In this study, the mean bias was higher for all estimating equations as BMI increased (**Fig 2**). No equation demonstrated better performance on agreement of GFR estimation with the reference mGFR over the others and the accuracy within 10% were unacceptably low among patients with high BMI extreme (BMI ≥35 kg/m^2^). Our findings were also observed in a recent study by Lopez-Martinez *et al*. [38] that the error from the most commonly used formulas including the MDRD and CKD-EPI equations was random and wide with 90% of estimates ranging from -50 to +50% of mGFR in obese patients with BMI ≥ 35 kg/m^2^. Although the adjustment for BSA is applied to all estimating equations in our study, the use of Du Bois formula which approximately underestimated the determination of BSA among obese Asian subjects by 1.3% [39], might yield an additional error on the GFR estimates from equations in patients with BMI ≥ 35 kg/m^2^. Another issue deserved to be considered is that indexing GFR with 1.73 m^2^ of BSA may mislead an interpretation of the reference mGFR result when considering on different types of weight used for the calculation of BSA as body size descriptor among obese subjects [40]. A previous study by Lemoine *et al*. [17] among 209 participants with mean BMI of 35 kg/m^2^ demonstrated that bias for a given estimating equation was greater for the reference mGFR scaled to BSA using real weight than ideal body weight and the accuracy of estimation of GFR compared to the reference mGFR indexed to real body weight seemed to be reduced for obese people with BMI greater than 40 kg/m^2^.

Taken together, we considered that all estimating equations, even modified with racial factor, failed to reflect the reference mGFR in obese patients. Direct measurement of GFR should be employed for confirmatory testing in some circumstances such as monitoring of GFR decline or the decision on initiation of renal replacement therapy among these patients.

Certain strengths of this study should be mentioned. We concomitantly assessed serum creatinine with the measurement of GFR on a relatively large sample of Asian obese subjects. We also collected ^99m^Tc-DTPA plasma clearance at 10 different time points. More repeated time-point plasma measurements after post-radioisotope injects can contribute to a better bi-exponential equation and help reduce error for the reference GFR measurement [20]. Furthermore, we used IDMS-traceable serum creatinine assay which is an essential part for improving the accuracy of GFR estimating equations [22]. However, we acknowledged that there was still low number of participants with morbid obesity in this study. Owing to the fact that serum creatinine is influenced by muscle mass whereas cystatin C has a direct relationship with fat mass [41], additional researches using alternative novel endogenous filtration markers such as beta-trace protein or β2-microglobulin might be needed for better accuracy of GFR estimating equations among people with obesity.

In conclusion, estimating equations generally underestimated the reference mGFR in subjects with obesity. The overall performance of GFR estimating equations demonstrated poor concordance with the reference mGFR among individuals with high BMI levels. In some clinical settings such as decision for dialysis initiation, the direct measurements of GFR are required to establish real renal function among obese population.
